# STRESSOR REACTIVITY DEPENDS ON THE CORTISOL AWAKENING RESPONSE AND REACTIVITY TO WORK OVERLOAD

**DOI:** 10.1093/geroni/igac059.2502

**Published:** 2022-12-20

**Authors:** MacKenzie Hughes, Christopher Hertzog, Shevaun Neupert, Scott Moffat

**Affiliations:** Georgia Institute of Technology, Atlanta, Georgia, United States; Georgia Institute of Technology, Atlanta, Georgia, United States; North Carolina State University, Raleigh, North Carolina, United States; Georgia Institute of Technology, Atlanta, Georgia, United States

## Abstract

Ecological momentary assessment (EMA) was used to understand the influence of individual differences in stress reactivity measured by the Perceived Stress Reactivity Scale (PSRS) and the cortisol awakening response (CAR) on emotional reactivity to stressors, operationalized as the within-person change in negative affect (NA) associated with stressor exposure. Five times per day for 10 days, 178 working adults ages 20-80 years old (M = 49.22, SD = 19.07) reported in EMAs their current NA and whether they had experienced a stressor since the previous survey. During the same period, participants provided seven salivary cortisol samples per day. Samples collected at awakening and 30-minutes post-awakening were used to calculate the CAR. Steeper CARs are hypothesized to have a role in preparing individuals to cope with upcoming daily demands. Before the EMA period, participants completed the PSRS, including its Work Overload Reactivity subscale. Multilevel models revealed a significant 3-way interaction between Stressor Exposure x CAR x Work Overload Reactivity predicting daily NA. Individuals with high Work Overload generally reported greater NA, regardless of stressor exposure or the magnitude of their CAR. Individuals with low Work Overload reported lower levels of NA on days they experienced more stressors than usual and had steeper CARs. Effects remained significant after controlling for neuroticism and the Perceived Stress Scale. Findings suggest the CAR’s potential role of preparing individuals for upcoming demands is moderated by work-related stress reactivity. Steeper CARs on days with more stressor exposure may provide enhanced emotional benefits for individuals low in workload reactivity.

